# Supplementary material for: Self-Regulation as a Mediator and Moderator Between School Stress and School Well-Being: A Multilevel Study
Source: Eur J Investig Health Psychol Educ. 2025 Dec 17;15(12):259. doi: 10.3390/ejihpe15120259 (PMC12731336; doi:10.3390/ejihpe15120259)
Supplement: Supplementary file 1 [file ejihpe-15-00259-s001.zip › ejihpe-3964652-supplementary.pdf]

Supplementary materials

Table S1. Correlations and parameters

Variable		n	%	Means (1)	(2)	(3)	(4)	Correlations (2)	(3)	(4)
Sex_1	Boy	336	48.5	-0.05	-0.20	0.14	-0.07			
Sex_2	Girl	341	49.3	-0.01	0.17	-0.10	0.09			
Sex_3	Another answer	15	2.2	0.34	0.65	-0.85	-0.49			
Class 4		321	46.4	-0.65	-0.23	0.14	0.40			
Class 6		251	36.3	0.20	0.11	-0.04	-0.26			
Class 8		120	17.3	1.20	0.38	-0.29	-0.51			
Class	ICC			-	0.08	0.06	0.16			
(1) Exposure to stressors	Class attribute, standardized 0-1	52						0.32a	-0.22	-0.38
(2) Perceived stress level	Student attribute, standardized 0-1	692						-	-0.48	-0.34
(3) Self-regulation (SR)	Standardized 0-1	692						-	-	0.45
(4) Well-being (WB)	Standardized 0-1	692						-	-	-

a – correlation for n=702
